# A network model of a multi-item working memory task based on competitive reverberating neural activity

**DOI:** 10.1186/1471-2202-12-S1-P83

**Published:** 2011-07-18

**Authors:** Joachim Haß, Daniel Durstewitz

**Affiliations:** 1 Bernstein-Center for Computational Neuroscience Heidelberg-Mannheim, Central Institute of Mental Health, Heidelberg University, Mannheim, 68159, Germany

## 

One of the greatest challenges for computational neuroscientists is to model the neural underpinnings of complex, ecologically relevant behavior. However, the construction of models for the neural dynamics which allow such behavior is very difficult, even when restricted to a single aspect of a task. Such a model needs to reproduce both the behavioral and the neuronal data, and should yet be simple enough to provide insight into the neural dynamics. Furthermore, to sufficiently constrain a model, electrophysiological recordings of awake, behaving animals are necessary, which are both challenging to obtain and to analyze.

Recently, researchers have investigated simultaneous multi-unit activity in the anterior cingulate cortex in rats during a spatial working memory and decision making task [1]. The animals were moving in an eight arm radial maze, foraging for food which was located at the end of each arm. In order to obtain food as fast as possible, the rats had to keep a memory of the arms they visited before, so they could avoid entering them again. The study reported clearly dissociable patterns of population activity that were specific for the different phases of the task [1]. Further analysis of this data showed that in a low-dimensional phase space projection, the multi-unit activity described recurrent, orbit-like trajectories which also distinguish between the different arms that the rats previously entered.

Here, we present a neural network model based on these observations which provides a possible neural basis for the multiple-item working memory needed in this task. The network consists of eight pools of neurons, which are connected in a circular fashion, such that activity continuously reverberates between states corresponding to ea ch of the eight arms. To encode whether a given arm was visited before or not without interrupting the flow of activity, each pool is subdivided into mutually inhibiting populations of neurons, which are equivalent in their projections from and to neighboring pools. One of these sub-populations is associated with a “new” arm, while the other one correspondingly encodes a previously visited, “old” arm. Finally, sensory stimulation is simulated by activating the “new” populations in the beginning of the trial, and the “old” population of a given arm at the time it is entered.

In order to perform the necessary computation, each pool must exhibit and maintain the appropriate bias between the two subpopulations, i.e. either the new or the old population must be active while the other is silent. This bias is initially induced by the sensory stimulation. After the stimulus is gone, the memory is maintained by the reverberating activity itself: Activity decays with a time constant such that there is still some fraction of the peak firing rate left by the time the activity wave returns to that pool. Thus, the population which is more active at this time will be enhanced faster and stronger by the wave, and the bias is fortified because of the mutual inhibition.

In terms of dynamics, the network gives rise to persisting working memory representations of the visited arms if the interplay between slowly decaying activity in each pool (which carries the selective bias), and reverberating activity forms a stable limit cycle in the phase space of firing rates. To confirm this quantitatively, we reduced the model further to a system of two pools and conducted a formal stability analysis on the resulting dynamics. In this way, we derived stability conditions on the parameters such that both selectivity between the new and old pools, and continuously reverberating activity is maintained. These conditions generalize to an arbitrary number of pools, and can thus be used to constrain an optimization process to fit the model to the trajectories in the neuronal data.
